# Effects of dangguijakyaksan on lower-extremity blood circulation disturbances in climacteric and postmenopausal women: Study protocol for a randomized, double-blind, placebo-controlled pilot trial

**DOI:** 10.1097/MD.0000000000017039

**Published:** 2019-09-13

**Authors:** Soo-Yeon Ahn, Seon-Eun Baek, Eun Ji Park, Hye-Won Kim, Jin-Ah Ryuk, Jeong-Eun Yoo

**Affiliations:** a Department of Obstetrics and Gynecology, College of Korean Medicine, Daejeon University; b Korean Medicine Department, Korea Institute of Oriental Medicine, Daejeon, Republic of Korea.

**Keywords:** blood circulation disturbance, climacteric, dangguijakyaksan, double-blind, herbal medicine, placebo-controlled trial, postmenopausal women, randomized trial, study protocol

## Abstract

**Background:**

Climacteric women experience various disorders, including hot flush, depression, insomnia, arthralgia, and hand and foot numbness. Dangguijakyaksan is among the most common treatments for climacteric syndrome, and its effect on depression, insomnia, hot flush and quality of life (QOL) in climacteric women has been reported multiple times. A recent animal study found dangguijakyaksan decreased serum lipid factors and improved blood circulation in a menopausal rat model; however, these effects have not been assessed in clinical trials. This study aims to assess the clinical effects and safety of dangguijakyaksan for lower-extremity blood circulation disturbances in climacteric women.

**Methods:**

This is a single-center, randomized, double-blinded, placebo-controlled pilot study that will be conducted at Dunsan Korean Medicine Hospital at Daejeon University. Forty-six climacteric women with lower-extremity blood circulation disturbances will be recruited and randomized (1:1) into either the dangguijakyaksan or placebo group. After 8 weeks of administration, the effects and safety of dangguijakyaksan will be assessed.

The primary outcome is the visual analogue scale for lower-extremity blood circulation disturbances, and it will be assessed on visits 1, 2, and 3. The secondary outcomes, Kupperman's index and blood deficiency scoring system, will be assessed on visits 1, 2, and 3, and accelerated photoplethysmography and digital infrared thermal imaging will be performed on visits 1 and 3. Moreover, blood lipid profile, follicle-stimulating hormone, and estradiol levels will be measured at the screening visit and visit 3. Blood tests will be performed at the screening visit and visit 3 to assess the safety of dangguijakyaksan. Statistical analysis will be performed using R-3.3.3 (Another Canoe), and within-group study variable differences after drug administration will be analyzed using paired t-test or Wilcoxon signed-rank test.

**Discussion:**

We expect to confirm the effects and safety of dangguijakyaksan on lower-extremity blood circulation disturbances in menopause, which would provide foundational data for planning subsequent studies.

## Abstract 2

Supplemental Digital Content is available in the text

## 1 Introduction

Climacteric women experience various climacteric disorders due to noticeable hormonal changes, including hot flush, depression, insomnia, arthralgia, and hand and foot numbness.^[1]^ According to the 2017 NICE guidelines, women aged 45 years and above who show climacteric signs, such as vasomotor symptoms and irregular menstruation patterns, can be diagnosed with climacteric syndrome without a hormone test.^[2]^ Many women experience discomfort associated with climacteric syndrome as reported by a study on climacteric women in five countries, including China and Taiwan, where about 92% of the women reported to have experienced climacteric symptoms.^[3]^

Hormone replacement therapy (HRT), the primary treatment for climacteric syndrome, is effective for vasomotor, reproductive, and urological symptoms and prevents bone loss and bone fractures. However, it increases the risk for cardiovascular disease and breast cancer and has several contraindications, including liver disease and hemostatic disorder. Thus, HRT is recommended to be used at a minimum dosage and period, and caution must be taken when applying it to in women aged above 60 years or in women whose menopause onset has reached at least 10 years.^[4]^

In fact, many climacteric women are confused about the implementation and continuance of HRT^[5]^; therefore, there are growing demands for studies on non-hormone therapies and complementary and alternative medicine.^[6,7]^

Dangguijakyaksan is a herbal medicine prescribed to individuals with low physical fitness, anemic tendency, hand and foot numbness, cold lower extremities, and climacteric disorder. Its effects on hot flush,^[8]^ insomnia,^[9]^ and depression^[10]^ in climacteric women have been reported by several clinical trials. A recent animal study reported that it lowered serum lipid levels and suppressed platelet coagulation and thrombosis in a menopausal rat model.^[11]^ As a translational research of this animal study, we aim to confirm whether dangguijakyaksan improves blood circulation via a similar action as estrogen on vascular endothelial cells since there is a lack of clinical trials examining its effects on climacteric women with lower-extremity blood circulation disturbances.

Thus, we designed a randomized, double-blinded, placebo-controlled study to randomize participants into either the dangguijakyaksan or placebo group at a 1:1 ratio to examine the effects and safety of dangguijakyaksan on the symptoms of lower-extremity blood circulation disturbances and explore the clinical trial feasibility.

## 2 Methods

### 2.1 Trial design

This is a single-center, randomized, double-blinded, placebo-controlled pilot study that will be conducted at Dunsan Korean Medicine Hospital at Daejeon University. After 8 weeks of administration, we will assess the effects and safety of dangguijakyaksan on lower-extremity blood circulation disturbances.

The study protocol was approved by the Institutional Review Board at Dunsan Korean Medicine Hospital at Daejeon University (DJDSKH-19-DR-07, date of approval: 2019.04.12), and was registered in the Clinical Research Information Service (CRIS), which is managed by the Korea Centers for Disease Control and Prevention (Registration number: KCT0003967, date of approval: 2019.05.22). In case of further revisions to the approved clinical trial, we will obtain approval for the revised protocol. The entire procedure of this study adheres to the Korean Good Clinical Practice (KGCP) and the *Declaration of Helsinki*.

### 2.2 Participants

#### 2.2.1 Inclusion criteria

The inclusion criteria are as follows:

(1) Age of 45 to 65 years
(2) Climacteric or postmenopausal women
  We will define climacteric women as those who meet one of the following criteria:
  - Premenopausal women who have had amenorrhea persisting for 3 to 12 months at the time of the screening visit
  - Women who have had irregular menstruation for the past 12 months from the date of the screening visit (change of menstrual cycle)
  We will define postmenopausal women as those who meet one of the following criteria:
  - Natural amenorrhea over the past 12 months
  - Six months of natural amenorrhea and blood FSH ≥ 40 mIU/mL
  - At least 6 weeks since bilateral oophorectomy (hysterectomy irrelevant)
  - Hysterectomy
(3) Lower-extremity blood circulation disturbances with one of the following symptoms:
  - Cold legs or feet
  - Leg numbness and cramps
  - Calf swelling and heaviness
(4) Individuals who gave written informed consent.

#### 2.2.2 Exclusion criteria

The exclusion criteria are as follows:

(1) Hormone therapy within 6 months of the screening visit
(2) Regular Korean medical treatment or other treatment considered pseudo-medical practice that may affect climacteric and menopause-related symptoms and has not passed the washout period designated by the researcher (acupuncture and moxibustion: at least one week; herbal medicine, herbal acupuncture, and agents with identical principle ingredients: at least 3 weeks)
(3) Use of health supplements or over-the-counter drugs to improve climacteric symptoms within 4 weeks of the screening visit
(4) HbA1c ≥ 7%
(5) Currently undergoing anti-cancer therapy after being diagnosed with a malignant tumor
(6) Behavioral disorder, depression, anxiety neurosis, or severe psychiatric disorder
(7) Aspartate transaminase (AST), alanine aminotransferase (ALT), alkaline phosphatase (ALP), and gamma-glutamyl transferase (GGT) 1.5 times above the normal upper limit
(8) Blood urea nitrogen (BUN), creatinine at 1.5 times above the normal upper limit
(9) Uncontrolled thyroid disease (individuals who are determined by the investigator as able to participate in the clinical trial may be included)
(10) Currently undergoing treatment for other diseases that may cause blood circulation disturbances (e.g., peripheral neuropathy and spinal disease)
(11) Currently undergoing blood transfusion or erythropoietin therapy for severe anemia
(12) Vaginal bleeding of unknown cause after menopause
(13) Endometrial cancer or endometrial hyperplasia
(14) Lactose intolerance
(15) Participation in another clinical trial with treatment intervention within the past three months
(16) Other cases considered inappropriate for participation as determined by the clinical trial investigator

### 2.3 Procedures

Forty-six climacteric women with lower-extremity blood circulation disturbance will be recruited through advertisements within and outside the hospital. The investigator will provide a thorough explanation about the purpose, procedure, intervention, benefits, and risks of the study as well as the ability to withdraw from the study at any time to the potential participants and obtain voluntary consent. Individuals who consent to participate will undergo a screening test, and their eligibility will be determined based on sociodemographic information, current disease, disease history, and drug history.

**Figure 1:**
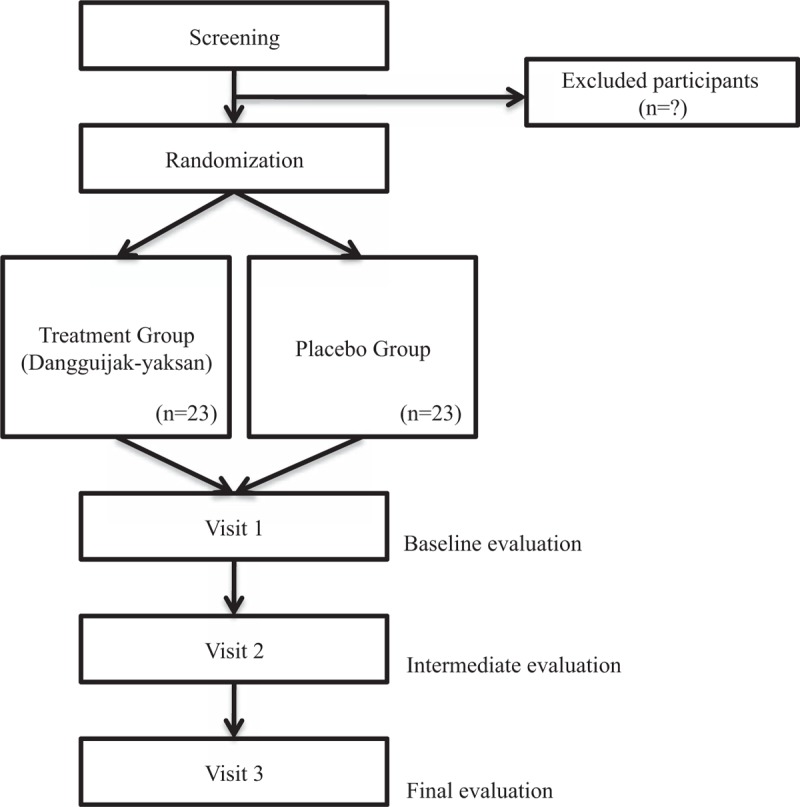
Study flow chart.

**Figure 2:**
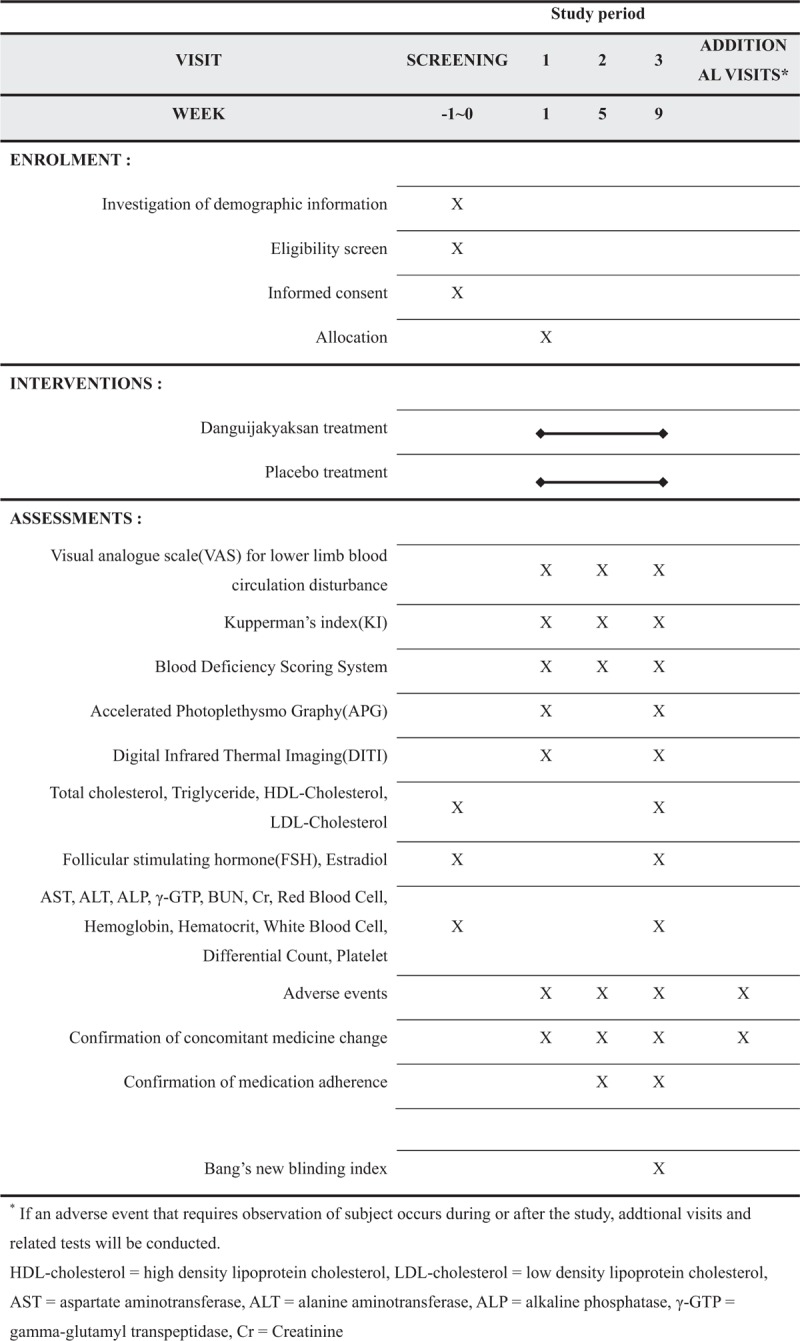
Schedule for the treatment and the outcome measurements.

Eligible participants will be randomized into either the dangguijakyaksan or placebo group in a 1:1 ratio. The participants will be on the treatments for 8 weeks, with double-blinding applied, and the planned surveys and tests will be performed at weeks 1, 5, and 9. (Figs. 1 and 2)

### 2.4 Interventions

The intervention group will take 3 g of dangguijakyaksan granules (Dangguijakyaksan Ext. 1.7 g) 3 times a day before or between meals for 8 weeks. Dangguijakyaksan consists of 6 herbs (i.e., Angelicae Gigantis Radix, Cnidii Rhizoma, Paeoniae Radix Alba, Poria cocos, Atractylodis Rhizoma Alba, Alismatis Rhizoma). The appropriate dose of each ingredient will be placed in the extractor with distilled water, filtered, and decompressed-concentrated to make 1.7 g of soft extract (Table 1). The dangguijakyaksan to be used in this study was extracted, concentrated, and dried according to the Korea Good Manufacturing Practice (KGMP) by Hanpoong Pharmaceuticals (Wanju, Republic of Korea).

**Table 1:**
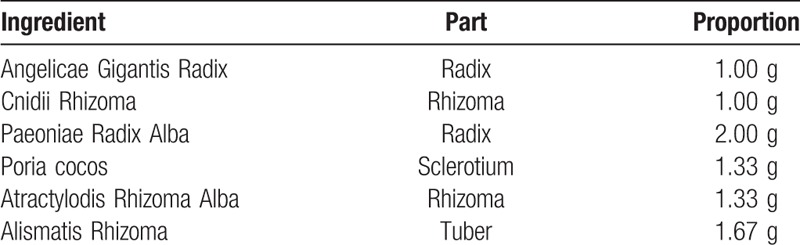
Composition of Dangguijakyaksan extract.

The control group will take 3 g of a placebo of the same form as the test drug using the same dosage. The placebo will contain lactose hydrate (1.476 g), corn starch (1.500 g), caramel coloring (0.014 g), and ginseng-flavor powder (0.010 g) and was prepared per the Granules section of the Korean Pharmacopoeia.

The test drug and placebo will be provided twice (week 1, week 5), and any unused drug and empty wrapping paper of the used drug will be retrieved to check participants’ medication adherence.

### 2.5 Concomitant drugs

During the clinical trial period, concomitant use of hormone therapy (estrogen, estrogen/progestogen, tibolone) that is related to climacteric syndrome, Korean medical treatment, health supplements, and over-the-counter drugs for the purpose of improving climacteric syndrome is prohibited.

Participants can continue on drugs that were been taken to treat chronic disease prior to the start of the clinical trial, preferably without changing the dose and type. If the drug dosage is changed, additional drugs are taken, or other treatments are received, the participants will report to the sub-investigator to record it in the case report form (CRF).

### 2.6 Study termination and withdrawal criteria

Study termination and withdrawal criteria are as follows.

(1) Participant or participant's legal representative withdraws consent to participate in the study
(2) A violation of the inclusion/exclusion criteria is discovered during the study
(3) Participant cannot continue the study due to a severe abnormal event
(4) Participant violates study protocol
(5) Participant is lost to follow-up
(6) Participant takes drugs, without instructions from his or her physician, which may impact the results of the study
(7) Other cases in which continuation of study is deemed inappropriate by the sub-investigator.

In addition, the study can be prematurely terminated for participants who no longer require additional drug administration due to loss of climacteric symptoms prior to completion of the 8-week intervention.

The investigator may indefinitely halt the study intervention at any point in the clinical trial in case of an abnormal event considered to cause harm to the participants. If the continuance of the intervention is later determined to indeed cause harm, the intervention will be permanently stopped. The clinical trial will be immediately permanently stopped for all participants who become pregnant.

### 2.7 Outcomes

All assessments will be performed by a blinded investigator per the planned schedule. The primary endpoint is the Visual Analogue Scale (VAS) value for symptoms of lower-extremity blood circulation disturbances. The secondary endpoints will be assessed using the Kupperman index (KI), blood deficiency scoring system, accelerated photoplethysmography (APG), digital infrared thermal imaging (DITI), blood lipid profile, follicular stimulating hormone (FSH), and estradiol (E2) levels.

#### 2.7.1 Primary outcome measure

*VAS for symptoms of lower-extremity blood circulation disturbances*: Under the supervision of the investigator, the participants will mark the perceived degree of their lower-extremity blood circulation deficiency symptoms on a 100-mm linear line, ranging from “no symptoms” to “most severe and intolerable symptoms,” and the investigator will read and record the value (mm) at the marked point. Assessment will be performed on weeks 1, 5, and 9.

#### 2.7.2 Secondary outcome measures

*KI*: The KI is widely used for the diagnosis of climacteric syndrome. It is classified into 6 domains (vasomotor disturbance, urological symptoms, systemic neurological symptoms, motor symptoms, digestive symptoms, and systemic symptoms) over 11 items.^[12]^ Each of the 11 items is rated on a four-point scale from 0 to 3, and the score of each item is weighted varyingly. Item 1 is given four points, items 2 to 4 are given 2 points, and items 5 to 12 are given 1 point. Assessment will be performed on weeks 1, 5, and 9.

*Blood deficiency scoring system*: We will use the blood deficiency scoring system in the Health Survey of the Department of Oriental Medicine at Toyama Medical and Pharmaceutical University, Japan. It consists of 17 items on 12 domains, including reduced concentration, insomnia sleep disorder, eye fatigue, and dizziness. Each item is answered with a yes or no, and a score of 30 or higher out of 100 is considered blood deficiency.^[13]^ Assessment will be performed on weeks 1, 5, and 9.

*APG (SA-6000)*: This is the second derivative of the waveform of digital plethysmography, which is a record of the changes in the finger blood volume through the arterial system. Since it is easy to measure and is sensitive to changes in arterial pressure, it is utilized in clinical practice to assess the progression of atherosclerosis and predict peripheral arterial disease or cerebrovascular disease or as a health index.^[14,15]^

Based on an assessment of the differential pulse wave index, stress power, blood vessel tension, and remained blood volume, APG is classified into stages 1 to 7, with a more advanced stage indicating a poorer waveform.^[16]^ Assessment will be performed on weeks 1 and 9.

*DITI (IRIS-XP,GOLD)*: DITI is a device that enables visual assessment and recording of body surface temperature. It can be used to safely examine abnormal muscle state and skin blood volume that affect skin temperature.^[17]^ Temperatures at ST32 and LR3 of the lower extremities will be compared. Assessment will be performed on weeks 1 and 9.

*Blood lipid profile (total cholesterol, triglyceride, HDL-cholesterol, LDL-cholesterol)*: An excessive level of LDL-C induces atherosclerosis by inducing endovascular inflammatory responses and forming plaques, which ultimately narrows blood vessels and disturbs blood flow. A high blood triglyceride level can also induce atherosclerosis since it can be easily converted to LDL-C. In contrast, a reduction in the HDL-C level actually increases the risk of atherosclerosis.^[18]^ Tests will be performed in a fasting state at the screening visit and on week 9.

*Follicle stimulating hormone (FSH), estradiol (E2)*: FSH is a hormone released from the pituitary that stimulates the growth of ovarian follicles and production of estrogen, and its concentration in the blood increases as a woman approaches menopause. Estradiol (E2) is an estrogen steroid hormone released in the ovaries that relatively accurately reflects ovarian functions, and its concentration is decreased at menopause^[19]^. Test will be performed at the screening visit and on week 9.

### 2.8 Safety

The sub-investigator will check for any changes in the concomitant drugs or treatments and record them at every visit. The sub-investigator will also measure the temperature, pulse, and blood pressure and check for adverse events at each visit.

To examine the safety of the drug, AST, ALT, ALP, GGT, BUN, creatinine, red blood cell, hemoglobin (Hb), hematocrit, white blood cell, differential count (segmented cell, monocyte, lymphocyte), and platelet tests will be performed at the screening test and on week 9. In the presence of abnormalities, factors that may impact the test will be checked (drinking, severe fatigue).

In women of childbearing age, a negative pregnancy test will be confirmed through a urine test prior to enrollment to the clinical trial, and patients with uncontrolled diabetes will be screened out through HbA1c testing at the screening visit.

The principal investigator (PI) will teach the sub-investigator and participants or their caregivers about all potential adverse events that may occur after the intervention. If a serious adverse event (SAE) occurs during the study period, the PI will orally report to the client and clinical research associate within 24 hours and submit a written SAE report within 7 days to the sponsor and Institutional Review Board to determine whether to continue or stop the clinical trial. Additional safety information will be periodically reported until the SAE is resolved.

If an unexpected adverse event occurs, even in participants who have completed the clinical trial, medical care will be provided at any time under the instructions of the PI or sub-investigator. The PI adheres to the Declaration of Helsinki in all matters pertinent to the study.

### 2.9 Sample size

This is a pilot study aiming to investigate the effects of dangguijakyaksan on lower-extremity blood circulation disturbances in climacteric and postmenopausal women. Given the lack of previous studies on blood circulation disturbances and dangguijakyaksan, we could not calculate the accurate sample size based on previous studies. However, considering the feasibility of this study, the sample size was set to 40, with each group containing 20 participants.

With reference to a recent clinical trial^[20]^ that investigated the effects of a herbal agent in climacteric women during 8 weeks like our study, the dropout rate was assumed to be 15%, and the final sample size was set to 46, with 23 in each group. 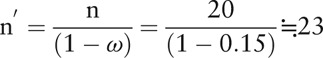


### 2.10 Randomization and allocation concealment

A statistician will create a randomization list using R-3.3.3 (Another Canoe), and block randomization will be performed. After creating a randomization list, the statistician will transfer the list to the investigator in charge of blinding, and the investigator will perform allocation to either the test drug or placebo group. The investigator in charge of blinding will then deliver the randomization table and group allocation information to the pharmaceutical personnel.

Once participants are enrolled, the coordinator will assign them an identification code (GY_1901-R1XXX), and participants will be allocated to each group at a 1:1 ratio.

The investigator will place an obscure envelope for an emergency that contains group allocation information inside. This emergency envelope will be turned over to the PI, and the PI will store it in a safe and accessible place.

### 2.11 Blinding

The test drug and placebo have been developed to have a nearly identical form and color and are packaged with the same packaging material. The participant identification code will be created such that it does not distinguish the intervention group from the control group. The participants, investigating Korean medical doctor, coordinator, managing pharmacist, monitoring staff, and statistician will only be provided with participant identification codes. The investigator in charge of blinding is the only person who will not be blinded, and the said investigator will not perform any roles in the study that require blinding.

Unblinding will only be considered when it is essential to know which treatment the participant is undergoing for the purpose of treating the participant. If unblinding is determined to be necessary, the PI or the Korean Institute of Oriental Medicine (KIOM) will be contacted first to obtain consent for the unblinding, and the blinding procedure is documented.

On visit 3, participants will be instructed to choose among “intervention group,” “control group,” or “I don’t know” regarding the group they had been placed in. This will be to calculate Bang et al's new blinding index^[21]^ for the assessment of the appropriateness of blinding.

### 2.12 Statistical methods

Statistical analyses will be performed using R-3.3.3 (Another Canoe), and missing values will be processed using the multiple imputation method. Continuous variables of social and demographic information will be presented as mean and standard deviation and analyzed using Student *t* tests (or Wilcoxon rank-sum test). Categorical variables will be presented as frequency and percentage and analyzed using Chi-square test (or Fisher exact test).

Analysis of covariance will be performed using the primary and secondary endpoint values at each time point as the dependent variables, baseline values as the covariates, and allocated group as the fixed factor. After dividing the study participants into either premenopausal or postmenopausal women, explorative subgroup analysis will be performed to examine whether the effects are identical for the two groups. Differences in the evaluation variables after taking the corresponding drug will be analyzed using paired *t* tests or Wilcoxon signed-rank test. The full analysis set (FAS) will be primarily used for analysis, and the per-protocol set (PPS) will be used adjunctively.

### 2.13 Data collection and management

At the screening visit, after obtaining informed consent, information on the duration of climacteric syndrome, medical background, and sociodemographic information will be collected. VAS values for symptoms of lower-extremity blood circulation disturbances, blood test results, body temperature test results, APG, and questionnaire responses will be recorded in the CRF. Various data and records related to the clinical trial will be safely stored at a designated place of storage.

Personal identifiable information will be kept confidential, and participants’ anonymity will be ensured even after publishing of the results of the clinical trial.

Participants’ information and blood collected during the clinical trial will only be used for research purposes. Participants’ identification codes, as opposed to their names, will be recorded in all documents related to the clinical trial, including the CRF.

### 2.14 Quality control

This clinical trial will be monitored to check whether protection of the participants’ rights and welfare and the reported clinical trial-related data are consistent, complete, and verifiable with the evidence documents and whether the clinical trial is being performed in adherence to the approved protocol and KGCP. Monitoring of the clinical trial will be performed by independent departments via regular visits or calls to the research institution.

## 3 Discussion

Dangguijakyaksan has been reported to be effective in improving sleep disorder,^[9]^ depression symptoms,^[10]^ hot flushes, and quality of life^[8]^ in climacteric women. Although the effects of dangguijakyaksan on blood circulation disturbances in climacteric have been confirmed in a rat model, there have been no relevant clinical trials.

The purpose of this pilot study is to investigate the efficacy and safety of dangguijakyaksan in improving symptoms of lower-extremity blood circulation disturbances in climacteric and postmenopausal women and explore the feasibility of clinical trial.

In this study, symptoms of lower-extremity blood circulation disturbances are broadly classified into “coldness,” where the lower abdomen or legs are cold, and “heaviness,” where the legs feel heavy and numb. VAS values, DITI, APG, and blood lipid profile (Total cholesterol, TG, LDL-cholesterol, and HDL-cholesterol) before and after treatment will be compared between the 2 groups to indirectly assess blood circulation disturbance changes. Furthermore, we also aim to observe changes in the Korean Medicine Pattern Identification by assessing the blood deficiency scoring system scores.

This study attempts to perform an objective assessment by comparing the changes in various parameters in addition to the participants’ subjective assessment. In addition, this study attempts to verify the effects of dangguijakyaksan in improving blood circulation, which has only been verified in an animal model, through a clinical trial. However, since this is a pilot study, it faces 1 limitation in that the sample size and study period is insufficient. Nevertheless, we expect this pilot study to provide basic data to aid in the planning and design of follow-up studies related to the effects of dangguijakyaksan in promoting blood circulation.

## Author contributions

**Conceptualization:** Jeong-Eun Yoo, Soo-Yeon Ahn, Jin-Ah Ryuk.

**Data curation:** Eun Ji Park, Hye-Won Kim.

**Formal analysis:** Seon-Eun Baek.

**Funding acquisition:** Jeong-Eun Yoo.

**Investigation:** Soo-Yeon Ahn, Hye-Won Kim.

**Methodology:** Jeong-Eun Yoo, Seon-Eun Baek.

**Project administration:** Soo-Yeon Ahn.

**Software:** Eun Ji Park.

**Supervision:** Jeong-Eun Yoo.

**Validation:** Seon-Eun Baek.

**Writing – original draft:** Soo-Yeon Ahn.

**Writing – review & editing:** Jeong-Eun Yoo.

Jeong-Eun Yoo orcid: 0000-0001-7087-1635.

## Supplementary Material

Supplemental Digital Content
